# Improving ^13^C-urea breath test performance metrics for diagnosis of *Helicobacter pylori* infection

**DOI:** 10.3389/fgstr.2025.1634183

**Published:** 2025-09-12

**Authors:** Paula Mantero, Germán Rodolfo Flekenstein, Mariana Andrea Janjetic, Julián Andrés Fuda, Horacio Emilio Torti, Gustavo Cernadas, Marcela Beatriz Zubillaga, Cinthia Gabriela Goldman

**Affiliations:** ^1^ Cátedra de Física, Facultad de Farmacia y Bioquímica, Universidad de Buenos Aires, Buenos Aires, Argentina; ^2^ Escuela de Nutrición, Facultad de Medicina, Universidad de Buenos Aires, Buenos Aires, Argentina; ^3^ Consejo Nacional de Investigaciones Científicas y Técnicas (CONICET), Buenos Aires, Argentina; ^4^ Centro de Investigación sobre Problemáticas Alimentarias y Nutricionales (CISPAN), Escuela de Nutrición, Facultad de Medicina, Universidad de Buenos Aires, Buenos Aires, Argentina; ^5^ Cátedra de Anatomía e Histología, Facultad de Farmacia y Bioquímica, Universidad de Buenos Aires, Buenos Aires, Argentina

**Keywords:** *Helicobacter pylori*, ^13^C-UBT, diagnosis, sensitivity, specificity, accuracy, cut-off

## Abstract

The ^13^C-Urea Breath Test (^13^C-UBT) is a popular, non-invasive method used for the diagnosis of *Helicobacter pylori* infection. This work evaluates its performance for the initial diagnosis and post-treatment follow-up in dyspeptic adults from Buenos Aires, Argentina. We retrospectively analyzed data from two earlier studies, which evaluated *H. pylori* infection using ^13^C-UBT and histology of gastric biopsies. Additionally, we assessed the ^13^C-UBT performance against the concordant results of both histology and PCR in a subsample with available data. The ^13^C-UBT was performed using a commercial kit, with isotope-ratio-mass-spectrometry (IRMS) as the measurement technique. Results from 154 volunteers were evaluated to assess the performance of ^13^C-UBT for the initial diagnosis of *H. pylori* infection, with histological evaluation as the reference method. For a cut-off value set at 3.5‰, sensitivity was 93.0%, specificity was 95.6%, accuracy was 94.2%, positive predictive value (PPV) was 96.4% and negative predictive value (NPV) was 91.5%. The subsample analysis of ^13^C-UBT vs. histology and PCR showed improved results: sensitivity 98.8%, specificity 98.3%, accuracy 98.6%, PPV 98.8% and NPV 98.3%. In contrast, ^13^C-UBT performance for confirming *H. pylori* eradication was studied in 46 patients, showing a sensitivity of 94.4%, specificity 100.0%, accuracy 97.8%, PPV 100.0% and NPV 96.6%. The analysis of sensitivity, specificity and accuracy as a function of the cut-off revealed that the optimal value could be lowered to 3.0‰ in our laboratory. These results demonstrate that the ^13^C-UBT is a non-invasive, highly accurate method for both the initial and post-treatment diagnosis of *H. pylori* infection.

## 1 Introduction

*Helicobacter pylori* is a Gram-negative, microaerophilic bacterium that affects, on average, about half of the world’s population (1, 2). This spiral-shaped microorganism colonizes the human gastric mucosa, causing chronic gastritis. Although most infected subjects are asymptomatic, approximately 10-15% may develop more severe pathologies such as dyspepsia, peptic ulcer disease, gastric cancer and mucosa-associated lymphoid tissue lymphoma (3).

Successful colonization and persistence in the hostile gastric environment are possible through different mechanisms. Flagellar motility, chemotaxis, adhesin expression, and immunomodulation allow *H. pylori* to migrate across the mucus layer from the stomach lumen to the gastric epithelium (4). Another crucial factor for *H. pylori* survival is the production of urease, an enzyme that hydrolyzes urea to carbon dioxide and ammonia, neutralizing gastric acidity. This change in pH both alters the mucus viscosity, which facilitates motility, and creates a gradient that helps *H. pylori* orient away from the lumen (3, 5).

The ^13^C-Urea Breath Test (^13^C-UBT) is a popular non-invasive method used for the initial diagnosis of *H. pylori* infection as well as for post-treatment eradication control (2, 6, 7). It is based on the urease activity of this microbe and involves the administration of ^13^C-labeled urea and the subsequent measurement of Carbon Isotope Ratio (CIR; ^13^CO_2_/^12^CO_2_ or Δ^13^CO_2_ per mil) in an exhaled breath sample, where ^13^CO_2_ enrichment over a cut-off value reflects the presence of *H. pylori*. Our research group has used this methodology in research projects (8–14) and technology transfer services in various clinical settings, assessing CIR by mass spectrometry.

Given the critical importance of local validation for diagnostic tests (7, 15), the aim of this study was to evaluate the performance of the ^13^C-UBT compared to histopathological diagnosis of *H. pylori* in adult patients with dyspepsia, both for initial diagnosis and post-treatment control in a population from Buenos Aires city, Argentina.

## 2 Method

We retrospectively analyzed data from a cross-sectional study and a longitudinal research project conducted by our laboratory (13, 14). Both protocols included dyspeptic adults (18–70 years) who were fasting overnight and had an indication for upper gastrointestinal (GI) endoscopy. Exclusion criteria included: active GI bleeding, prior GI surgery, neoplastic disease, diabetes, celiac disease, thyroid, renal, or hepatic pathologies, drug abuse, coagulopathies, pregnancy, previous *H. pylori* treatment, and use of antimicrobials or acid suppressants 4 weeks before enrollment. All volunteers signed a written informed consent. These protocols were approved by the Ethics Committee of the participating institutions and were performed in accordance with the principles of the Declaration of Helsinki and the Guidelines for Good Clinical Practice.

*H. pylori* status was evaluated by ^13^C-UBT and histological examination of gastric biopsies. The ^13^C-UBT was performed using a commercial kit (TAU-KIT, Isomed Pharma S.L., Madrid, Spain), as previously described (14). Basal breath samples were collected 10 minutes after ingestion of 100 mL citric acid solution (17.2 g/L). Then, 50 mL of an aqueous solution containing 100 mg ^13^C-labeled urea was administered and post-^13^C-urea breath samples were obtained after 30 minutes, according to the manufacturer’s specifications. CIR was measured in our laboratory using an isotope-ratio mass spectrometer (IRMS) coupled with a gas chromatograph (Finnigan-MAT GmbH, ThermoQuest Corp., Bremen, Germany) and subsequently compared to an international standard for carbon isotopic composition (VPDB) to calculate the delta 13 PDB (Del 13 PDB). The parameter used to assess ^13^CO_2_ enrichment was the Delta Over Baseline (DOB), calculated as the algebraic difference between the Del 13 PDB in the post-^13^C-urea samples and the basal samples, with a cut-off value set at 3.5‰ for *H. pylori* infection diagnosis (6, 16).

The endoscopic procedure was performed 1 to 2 hours after the ^13^C-UBT. A total of four gastric biopsies were obtained, with two samples collected from the antrum and two from the body. One biopsy from each site was used for histological examination. Briefly, “biopsies were processed by formalin immersion for 2 h, dehydration in 96% ethanol for 6 h, 100% ethanol for 4 h and xylene for 3 h, with immersion in paraffin at 56-58°C for 3 h and at 62°C for 3 h. Consecutive 4 μm sections were obtained using a spin tissue processor (MicromSTP120, ThermoScientific Corp., Walldorf, Germany) for hematoxylin-eosin and Giemsa histologic staining” (14). Microscopic assessment diagnosed *H. pylori* infection if curved rods were identified in one or both samples. The two remaining biopsies were reserved for molecular analysis by polymerase chain reaction (PCR). Bacterial DNA was isolated from the biopsies using the QIAamp Mini Kit (QIAGEN, INC., CA, United States). *H. pylori* infection was evaluated by amplification of the s-region of the *vacA* gene, which is present in all *H. pylori* strains. Primers va1F (5’-ATGGAAATACAACAAACACAC-3’) and va1XR (5’-CCTGAGACCGTTCCTACAGC-3’) were used, yielding a product of 176 bp for *vacA* s1 allele and 203 bp for type s2 variants (17). The PCR mixture contained 1X Taq polymerase buffer, 1.5 mM MgCl_2_, 0.2 mM each deoxynucleotide, 1.0 U of Platinum^®^ Taq DNA Polymerase (Invitrogen Argentina, Buenos Aires, Argentina), 0.1 μg each oligonucleotide primer, and 5 μL of DNA template in a total volume of 50 μL. PCR incubation was performed in an automatic thermocycler (MyCycler, BioRad, CA, United States) with an initial denaturation for 3 min at 94°C, followed by 35 cycles of 30 s at 94°C, 45 s at 50°C, and 45 s at 72°C, and a final extension at 72°C for 5 min. A 10 μL aliquot was analyzed by electrophoresis on a 1.5% agarose gel stained with ethidium bromide, and the PCR products were visualized under UV light, as previously described (13, 14).

The sensitivity, specificity, accuracy, positive predictive value (PPV) and negative predictive value (NPV) of the ^13^C-UBT for initial diagnosis were calculated from the cross-sectional study data using Giemsa staining histology as a reference method and concordant histology and PCR results in a sub-sample set. Meanwhile, the results from the longitudinal protocol allowed us to estimate these same parameters for the ^13^C-UBT as a post-treatment control diagnostic method. We further evaluated these performance metrics by setting different cut-off points ranging from 1.5‰ to 5.0‰ to include the gray zone associated with inconclusive ^13^C-UBT results (6, 16, 18). We used the GraphPad Prism (Version 8.0.1, December 2018, Boston, Massachusetts, United States) to compute the 95% confidence interval (CI95%) of the proportions through the hybrid Wilson/Brown method (19, 20).

## 3 Results

Data from 163 volunteers enrolled between 2012 and 2015 (13) were processed to evaluate the ^13^C-UBT for the initial diagnosis of *H. pylori* infection. We excluded 9 patients lacking the histology report. The median age of the included participants was 40.0 years (IQR, 27.8-51.0 y), 58.4% (CI95%, 50.5%-65.9%) were female, and the prevalence of *H. pylori* infection, as estimated by histopathology, was 55.8% (CI95%, 48.0%-63.5%). The test performance characteristics were calculated for a cut-off value of 3.5‰, as is usually set in our laboratory. The results were as follows: sensitivity 93.0% (CI95%, 85.6%-96.8%), specificity 95.6% (CI95%, 87.8%-98.8%), accuracy 94.2% (CI95%, 89.3%-96.9%), PPV 96.4% (CI95%, 89.9%-99.0%) and NPV 91.5% (CI95%, 82.8%-96.1%).

**Figure 1:**
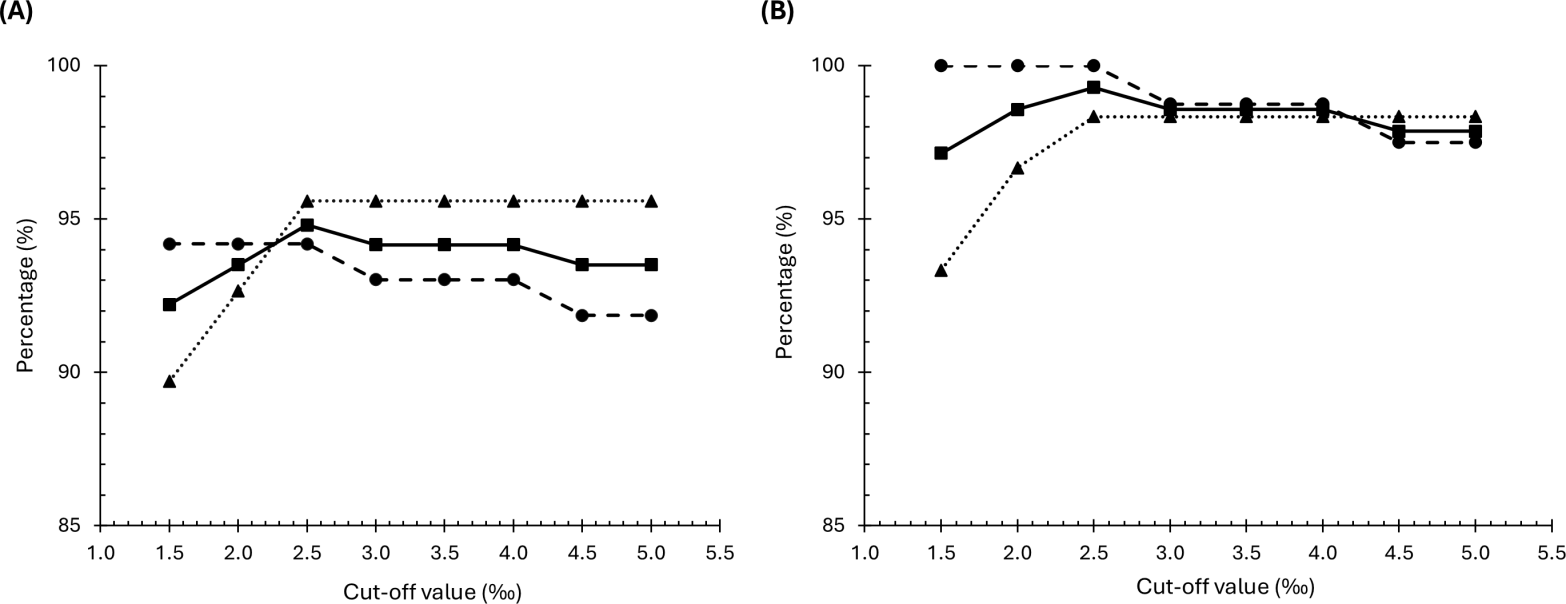
Sensitivity (•), specificity (▴) and accuracy (▪) of ^13^C-UBT at different cut-off values for initial diagnosis of *H. pylori* infection compared to histology **(A)** and concordant histology and PCR **(B)**.

The DOB results allowed us to assess the test performance for different cut-off values, ranging from 1.5‰ to 5.0‰. **Table 1** summarizes the sensitivity, specificity, accuracy, PPV, and NPV calculated for each cut-off. A cut-off of 2.5‰ maximized all these parameters, resulting in the best performance for the test, as shown in **Figure 1A**.

**Table 1:** Sensitivity, specificity, accuracy, positive predictive value (PPV) and negative predictive value (NPV) of ^13^C-UBT vs. histology at different cut-off values for initial *H. pylori* diagnosis (*n* = 154).

Cut-off DOB (‰)	True positive (*n*)	True negative (*n*)	False positive (*n*)	False negative (*n*)	Sensitivity (%)	Specificity (%)	Accuracy (%)	PPV (%)	NPV (%)
1.5	81	61	7	5	94.2	89.7	92.2	92.0	92.4
2.0	81	63	5	5	94.2	92.6	93.5	94.2	92.6
2.5	81	65	3	5	94.2	95.6	94.8	96.4	92.9
3.0	80	65	3	6	93.0	95.6	94.2	96.4	91.5
3.5	80	65	3	6	93.0	95.6	94.2	96.4	91.5
4.0	80	65	3	6	93.0	95.6	94.2	96.4	91.5
4.5	79	65	3	7	91.9	95.6	93.5	96.3	90.3
5.0	79	65	3	7	91.9	95.6	93.5	96.3	90.3

Given the limitations of histology as an exclusive diagnostic method for *H. pylori* infection, we analyzed the performance of the ^13^C-UBT by comparing it to concordant histology and PCR results in our cross-sectional study. One of the patients lacked PCR results, and 13 had non-concordant results between histology and PCR: specifically, 8 individuals had a negative *H. pylori* diagnosis by histology but a positive PCR result, while 5 were considered positive by histology with no amplification by PCR. This population of 140 adults had a median age of 41.0 years (IQR, 27.0-51.0 y), 57.1% (95% CI, 48.9%-65.0%) were female and *H. pylori* infection had a prevalence of 57.1% (95% CI, 48.9%-65.0%).

The test performance metrics of ^13^C-UBT against the combined histology and PCR reference were improved compared to the metrics when compared against histology alone. The calculated sensitivity was 98.8% (95% CI, 93.3%-99.9%), specificity was 98.3% (95% CI, 91.1%-99.9%), accuracy was 98.6% (95% CI, 94.9%-99.7%), PPV was 98.8% (95% CI, 93.3%-99.9%) and NPV was 98.3% (95% CI, 91.1%-99.9%). These parameters were also higher when calculated at different cut-off values, as shown in **Table 2**. Consistent with the previous analysis, the best accuracy was observed at a cut-off value of 2.5‰ (**Figure 1B**).

**Table 2:** Sensitivity, specificity, accuracy, positive predictive value (PPV) and negative predictive value (NPV) of ^13^C-UBT vs. concordant histology and PCR at different cut-off values for initial *H. pylori* diagnosis (*n* = 140).

Cut-off DOB (‰)	True positive (*n*)	True negative (*n*)	False positive (*n*)	False negative (*n*)	Sensitivity (%)	Specificity (%)	Accuracy (%)	PPV (%)	NPV (%)
1.5	80	56	4	0	100.0	93.3	97.1	95.2	100.0
2.0	80	58	2	0	100.0	96.7	98.6	97.6	100.0
2.5	80	59	1	0	100.0	98.3	99.3	98.8	100.0
3.0	79	59	1	1	98.8	98.3	98.6	98.8	98.3
3.5	79	59	1	1	98.8	98.3	98.6	98.8	98.3
4.0	79	59	1	1	98.8	98.3	98.6	98.8	98.3
4.5	78	59	1	2	97.5	98.3	97.9	98.7	96.7
5.0	78	59	1	2	97.5	98.3	97.9	98.7	96.7

We also evaluated the performance of the ^13^C-UBT for the post-treatment control of *H. pylori* after eradication treatment. This analysis was based on data from a longitudinal study conducted between 2015 and 2017 (14), which included 46 subjects who were initially diagnosed with the infection, underwent antimicrobial therapy, and subsequently returned for follow-up. The median age of this group was 47.5 (IQR, 35.8-55.3) years, and 63.0% (95% CI, 48.6%-75.5%) were female. Based on histopathology, the prevalence of *H. pylori* infection was 39.1% (95% CI, 26.4%-53.5%).

**Figure 2:**
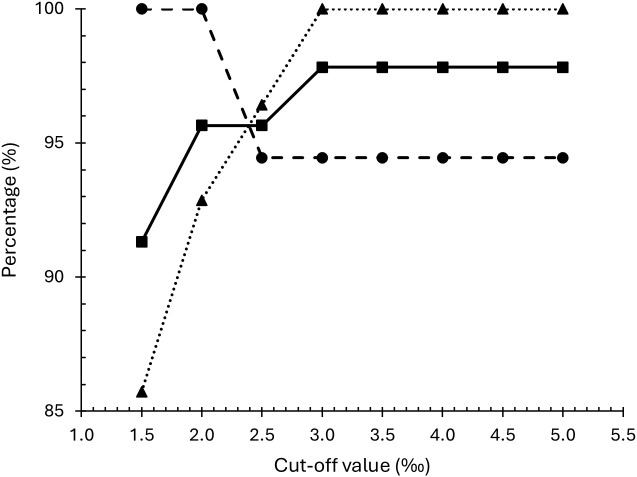
Sensitivity (•), specificity (▴) and accuracy (▪) of ^13^C-UBT at different cut-off values for *H. pylori* infection control after-treatment compared to histology.

In this context, the ^13^C-UBT showed a sensitivity of 94.4% (95% CI, 74.2%-99.7%), a specificity of 100.0% (95% CI, 87.9%-100.0%), an accuracy of 97.8% (95% CI, 88.7%-99.9%), a PPV of 100.0% (95% CI, 81.6%-100.0%) and a NPV of 96.6% (95% CI, 82.8%-99.8%). These parameters were also calculated at different cut-off values, as detailed in **Table 3**. Under these circumstances, a value of 3.0‰ was the lowest cut-off to achieve 100% specificity and PPV, with sensitivity, accuracy and NPV all exceeding 94%. At a cut-off of 2.5‰, both specificity and accuracy decreased without improving sensitivity. Further reductions in the cut-off resulted in a sensitivity of 100%, but a sharp decline in specificity (**Figure 2**).

**Table 3:** Sensitivity, specificity, accuracy, positive predictive value (PPV) and negative predictive value (NPV) of ^13^C-UBT vs. histology at different cut-off values for *H. pylori* post-treatment follow-up (*n* = 46).

Cut-off DOB (‰)	True positive (*n*)	True negative (*n*)	False positive (*n*)	False negative (*n*)	Sensitivity (%)	Specificity (%)	Accuracy (%)	PPV (%)	NPV (%)
1.5	18	24	4	0	100.0	85.7	91.3	81.8	100.0
2.0	18	26	2	0	100.0	92.9	95.7	90.0	100.0
2.5	17	27	1	1	94.4	96.4	95.7	94.4	96.4
3.0	17	28	0	1	94.4	100.0	97.8	100.0	96.6
3.5	17	28	0	1	94.4	100.0	97.8	100.0	96.6
4.0	17	28	0	1	94.4	100.0	97.8	100.0	96.6
4.5	17	28	0	1	94.4	100.0	97.8	100.0	96.6
5.0	17	28	0	1	94.4	100.0	97.8	100.0	96.6

## 4 Discussion

The ^13^C-UBT is a safe, non-invasive and widely used diagnostic method with high sensitivity and specificity for the initial diagnosis of *H. pylori* infection as well as for the control after eradication treatment (2). Local validation is recommended as demographic characteristics may vary across populations, and several test parameters can affect its outcome, including the ^13^C-urea dose, the chosen cut-off value, the sampling time after ^13^C-urea administration, the type of test meal and the measuring equipment (7). Based on results from two previous studies conducted by our research group, we evaluated the performance of ^13^C-UBT in patients from Buenos Aires city using a commercial kit (TAU-KIT).

The test demonstrated a robust performance, both for the initial diagnosis of *H. pylori* infection and for confirming eradication after treatment, under the conditions used in our study. These conditions included initial citric acid intake, a 100 mg ^13^C-urea dose in an aqueous solution, 30 minutes sampling time after urea ingestion, assessment using IRMS, and 3.5‰ cut-off value. However, our analysis of sensitivity, specificity and accuracy at different cut-off values -when evaluating ^13^C-UBT against histology alone and the combined concordant results of histology and PCR- revealed that for initial diagnosis of *H. pylori* infection the cut-off could be lowered to 2.5‰. This adjustment increased the sensitivity without compromising the specificity, thus improving accuracy. Further reducing the cut-off value, however, did not result in any additional increase in sensitivity but caused a drop in the specificity. In contrast, for post-treatment control, a 3.0‰ cut-off provided the best accuracy; lowering it to 2.5‰ maintained sensitivity but led to a decrease in specificity, thereby reducing accuracy. These outcomes led us to select 3.0‰ as the most appropriate cut-off value for use in our experimental setup.

It is important to highlight that, in this study, these test performance metrics were initially estimated using histology as the gold standard, even though it is widely known that it has both advantages and disadvantages, as any other reference method. In this case, histology limitations may be related to the patchy distribution of *H. pylori* and the varying bacterial density within the gastric mucosa. This can result in a sampling error, as well as interobserver variability due to differences in the pathologists’ expertise in identifying the microorganism (21). Despite these limitations, ^13^C-UBT performance values for initial diagnosis of *H. pylori* infection observed in this study are consistent with those reported in systematic reviews and meta-analyses of this technique (22). Furthermore, performance metrics of the ^13^C-UBT improved even more when we used two coincident diagnostic methods, histology and PCR, as a gold standard (7, 23).

A key strength of our study was that it also evaluated the ^13^C-UBT for post-treatment assessment, which yielded enhanced performance metrics. In this analysis, the reference method was restricted to histology due to a lack of PCR results for many participants in this cohort, which we acknowledge as a potential limitation.

Our investigation enrolled adult dyspeptic patients from the urban region of Buenos Aires. The prevalence of *H. pylori* infection within this study population aligns with rates reported for symptomatic individuals in urban areas of other Argentine provinces (24–29), thus supporting the broader applicability of our optimized cut-off to these demographically similar groups. Nevertheless, the optimized ^13^C-UBT cut-off may not be universally applicable. Its utility could be limited in other Latin American populations exhibiting higher *H. pylori* prevalences (30, 31), or in the diagnosis of infection in pediatric cases, where variables such as a reduced ^13^C-urea dose could impact DOB values.

The relevance of improving the performance metrics of the ^13^C-UBT lies in its ability to avoid both missed infections and unnecessary antibiotic exposure. Reducing false negative results may prevent the progression of gastrointestinal pathologies associated with *H. pylori* infection that could lead to the development of gastric cancer, with the resulting decrease in healthcare costs related to severe outcomes. Conversely, lowering false-positive results may reduce antibiotic misprescription, thereby mitigating the emergence of antimicrobial resistance (2).

This study shows that the ^13^C-UBT is highly sensitive and specific for both the initial diagnosis and for the post-treatment assessment of *H. pylori* infection, with its only drawback being the economic cost. The high accuracy of this non-invasive test, together with its simplicity and its capacity to detect active infection by evaluating the entire gastric mucosa, makes it a method of choice whenever the technology is available.

## Data Availability

The data analyzed in this study is subject to the following licenses/restrictions: No restrictions are applied to the dataset. Requests to access these datasets should be directed to CG, cgold@ffyb.uba.ar.
